# Phylogenetic analysis of *Leuconostoc* and *Lactobacillus* species isolated from sugarcane processing streams

**DOI:** 10.1002/mbo3.1065

**Published:** 2020-06-04

**Authors:** Sanet Nel, Stephen B. Davis, Akihito Endo, Leon M. T. Dicks

**Affiliations:** ^1^ Sugar Milling Research Institute NPC c/o University of KwaZulu‐Natal Durban South Africa; ^2^ Department of Microbiology Stellenbosch University Stellenbosch South Africa; ^3^ Department of Food, Aroma and Cosmetic Chemistry Tokyo University of Agriculture Hokkaido Japan

**Keywords:** dextran, *Lactobacillus*, *Leuconostoc*, phylogenetic analysis, sugarcane

## Abstract

High levels of gums such as dextran, produced by *Leuconostoc* and *Lactobacillus* spp., have a severe impact on factory throughput and sugar quality. This study aimed to determine the phylogenetic relationships between gum‐producing *Leuconostoc* and *Lactobacillus* bacteria which were isolated from various locations in a sugarcane processing factory at times when low‐ and high‐dextran raw sugar, respectively, were produced. Phylogenetic analysis of 16S rRNA gene sequences grouped 81 isolates with the type strains of *Leuconostoc mesenteroides* (subspp. *mesenteroides*, *dextranicum,* and *cremoris*), *Leuconostoc pseudomesenteroides, Leuconostoc lactis,* and *Leuconostoc citreum*, respectively. Forty‐three isolates clustered with the type strain of *Lactobacillus fermentum*. The phylogenetic relatedness of the isolates was determined by sequencing and analysis of the housekeeping genes *rpoA* and *dnaA* for *Leuconostoc* spp. and the *pheS* and *tuf* genes for the *Lactobacillus* spp. The *rpoA* gene proved discriminatory for the phylogenetic resolution of all of the isolated *Leuconostoc* spp. and the *dnaA* housekeeping gene was shown to be effective for isolates clustering with the type strains of *Leuc. mesenteroides* and *Leuc. citreum*. None of the loci examined permitted differentiation at the subspecies level of *Leuc. mesenteroides*. Single‐locus analysis, as well as the concatenation of the *pheS* and *tuf* housekeeping gene sequences, yielded identical phylogenies for the *Lactobacillus* isolates corresponding to *L. fermentum*.

## Graphical abstract

This study aimed to determine the phylogenetic relationships between gum‐producing *Leuconostoc* and *Lactobacillus* strains isolated from various locations in a sugarcane processing factory at times when sugars with a low‐ and high‐dextran content were produced.

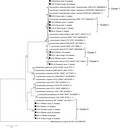

## 1 INTRODUCTION

Problems encountered with microbial degradation of harvested sugarcane, followed by further spoilage during processing, leads to a poor‐quality sugar and severe economic losses. In the sugar industry, microbial exopolysaccharides such as dextran are also referred to as “gums.” Spoilage bacteria in sugarcane processing have historically been identified by phenotypic methods, which failed to accurately differentiate between the genera *Leuconostoc* and *Lactobacillus* and between species within the genus *Leuconostoc* (Maniatis, Fritsch, &Sambrook, 1982). The last attempt at the profiling of spoilage bacteria in a sugarcane processing factory was more than 30 years ago. In that study, Lillehoj, Clarke, and Tsang (1984) identified *Leuconostoc mesenteroides* as the dominant species in factory processing streams. The authors differentiated *Leuconostoc* from *Lactobacillus* based on the assumption that lactobacilli do not produce dextran from sucrose, despite reports to the contrary (Duncan & Seeley, 1963, 1965; Pederson & Albury, 1955). Previous attempts by sugar technologists to identify spoilage bacteria in sugarcane processing factories have all been hampered by the lack of available identification methods of high discriminatory power.

Dextran‐producing strains of *Leuconostoc mesenteroides* have been implicated in the reduction of factory throughput and quality of the produced sugar (Eggleston, Morel du Boil, & Walford, 2008). Some strains of *Leuc. mesenteroides* produce as much as one‐part dextran from 40 parts of sucrose after only 6 hr (Cerutti de Guglielmone, Diez, Cárdenas, & Oliver, 2000). Apart from an increase in viscosity, other metabolites produced during the degradation of sucrose reduce the purity of the cane juice and thus also sucrose recovery. Impure juice requires longer boiling times, leading to higher sucrose inversion losses. The impurities reduce evaporation rates, and sugar crystals take longer to form (Godshall, Legendre, Clarke, Miranda, & Blanco, 1996; Jimenez, 2005). Dextran shows high (20%) transfer from juice to crystal, resulting in high carryover from the factory to the refinery. Refiners and buyers of raw sugar prefer the product to have low levels of dextran (<100–150 mg/kg), even if the purchase contract does not specify dextran content (Ravnö & Purchase, 2005). Economic losses due to microbial activities are, therefore, not limited to the direct loss of recoverable sucrose and indirect loss due to reduced factory throughput, but also finding financially attractive markets for high‐dextran raw sugar (Moodley & Khomo, 2018).

A recent study by Nel, Davis, Endo, and Dicks (2019a) used phylogenetic analysis of 16S rRNA gene sequences for the profiling of gum‐producing bacteria which were isolated from various locations in a South African sugarcane processing factory at times when low‐ and high‐dextran raw sugar, respectively, were produced. Although 16S rRNA gene sequences have been widely used as a phylogenetic marker in bacterial taxonomy, the method has limitations and is not reliable in distinguishing species and subspecies with high sequence similarities (Jeon et al., 2017). *Leuc. mesenteroides* and *Leuconostoc pseudomesenteroides* are good examples. The two species share almost identical 16S rRNA gene sequences, with differences in only 5 of the 1,483 nucleotides (Martinez‐Murcia & Collins, 1990). Comparisons among housekeeping gene sequences are commonly used to overcome the limitations of 16S rRNA coding region sequencing (Chelo, Ze‐Ze, & Tenreiro, 2007; De Bruyne et al., 2007; Naser et al., 2007; Yu et al., 2012). Single protein‐coding genes do not reflect general phylogenetic relationships due to potential horizontal gene transfer (HGT) or lateral gene transfer (Gogarten, Doolittle, & Lawrence, 2002; Macheras et al., 2011). Multiple gene‐based phylogenies were introduced which have been used more frequently to overcome the bias caused by single gene sequence‐based phylogenies (Glaeser & Kämpfer, 2015). Concatenation of several housekeeping genes may reduce the weight of HGT, and it could accurately locate taxonomic positions for closely related species and strains (Glaeser & Kämpfer, 2015).

Ricciardi, Storti, Zotta, Felis, and Parente (2020) described a polyphasic approach for the identification of *Leuc. mesenteroides* at the species and subspecies level. This approach was based on species‐specific PCR and multiplex PCR and was successful for the rapid identification of *Leuc. mesenteroides* strains, and it provided a reliable separation among *Leuc. mesenteroides* subsp. *mesenteroides* and *Leuc. mesenteroides* subsp. *cremoris*. However, it did not resolve the ambiguities in the identification of some strains of *Leuc. mesenteroides* subsp. *dextranicum* and *Leuc. mesenteroides* subsp. *jonggajibkimchii*. Ricciardi et al. (2020) suggested that the genome‐based classification of *Leuc. mesenteroides* should be supported by comparative metabolic diversity studies to identify molecular markers, such as taxonomically and functionally relevant genes, for rapid detection and discrimination of strains.

Previously, we reported on the phylogenetic identification of 430 gum‐producing isolates from harvested sugarcane and sampled from a South African sugarcane processing factory, based on partial 16S rRNA gene sequence analysis (Nel et al., 2019a). A large number of these isolates (47%) were identified as *Weissella cibaria* and *Weissella confusa*, respectively (Nel, Davis, Endo, & Dicks, 2019b). Most of these bacteria were isolated from the prepared (shredded) sugarcane at a time when high‐dextran raw sugar was produced. The only bacteria isolated from the juice screen and the mixed juice tank (24% of the isolates) were identified as *Bacillus amyloliquefaciens* and *Bacillus subtilis*, respectively (Nel, Davis, Endo, & Dicks, 2019c). Again, most of these bacilli were isolated when high concentrations of dextran were reported in the raw sugar. Only 19% of the isolates were identified as *Leuconostoc* spp. and 10% as *Lactobacillus* spp. based on initial 16S rRNA gene sequencing analysis (Nel et al., 2019a). This study aimed to determine the phylogenetic relationships between the isolated *Leuconostoc* and *Lactobacillus* bacteria based on the phylogenetic relatedness of selected housekeeping gene sequences.

## 2 MATERIALS AND METHODS

### 2.1 Isolation of gum‐producing bacteria

Samples of shredded (prepared) sugarcane and samples from the diffuser sump, juice screen (Dutch State Mines; DSM screen), mixed juice tank (MJ tank), filtrate, mud trough, and syrup tank in a South African sugarcane processing factory were collected and screened for the presence of gum‐(polysaccharide) producing bacteria (Nel et al., 2019a). Once‐off samples at each sampling location were taken during September 2013, when low‐dextran concentrations (<70 ppm) in the produced raw sugar were reported. This was repeated in November 2013, when high‐dextran concentrations (>500 ppm) in raw sugar were found. Cane samples (10 g each) were added to 100 ml phosphate‐buffered saline (PBS; Green & Sambrook, 2012) and incubated on a rotary shaker (30°C, 150 rpm) for 1 hr. Liquid samples collected from each of the sampling points and PBS‐cane suspensions were serially diluted in PBS. Serial dilutions were streaked onto modified dextransucrase‐inducing agar with the following composition: sucrose 100 g/L, peptone 20 g/L, KH_2_PO_4_ 20 g/L, agar 15 g/L, and R‐salts (4% MgSO_4_·7H_2_O, 4% NaCl, 0.2% FeSO_4_·7H_2_O, and 0.2% MnSO_4_·H_2_O) 5 ml (Tsuchiya et al., 1952). Plates were incubated at 30°C for 14–18 hr. Visual screening of colonies with a glistening and slimy appearance on the dextransucrase‐inducing medium was carried out to select for gum‐producing bacteria. A total of 430 colonies were isolated and streaked to purity on modified dextransucrase‐inducing agar. From these plates, a single colony was inoculated into 5 ml MRS broth (Biolab, Merck South Africa), and the cultures were incubated on a shaking incubator (150 rpm) for 14–18 hr at 30°C. Cells were harvested by centrifugation (16,000 *g*, 25°C, 2 min), resuspended in sterile glycerol (200 μl; 50%, v/v), and stored at −70°C.

### 2.2 Genomic DNA extraction

An aliquot of ten microliters from stock cultures of previously isolated (Nel et al., 2019a) *Leuconostoc* (81 isolates) and *Lactobacillus* (43 isolates) was inoculated into 5 ml sterile MRS broth (Biolab, Merck) and incubated for 16 hr at 30°C on a rotary shaker (150 rpm). Cells were harvested (16,000 *g*, 25°C, 2 min) and genomic DNA extracted using the GeneJET Genomic DNA Purification kit (Thermo Scientific, Inqaba Biotechnical Industries) according to the manufacturer's instructions. Purified DNA was suspended in 50 μl elution buffer and used as a template in amplification reactions.

### 2.3 Amplification of the 16S rRNA genes, and housekeeping genes *rpoA, dnaA*, *pheS*, and* tuf*

Genomic DNA was used as a template to amplify sequences of the 16S rRNA genes of all species, *rpoA* and *dnaA* genes of *Leuconostoc* spp., and *pheS* and *tuf* genes of *Lactobacillus* spp. using the primers listed in Table 1. Reactions were carried out in 50 μl, containing 10 pmol of each primer, 200 µM of each deoxynucleoside triphosphate (Thermo Scientific), 10 µl of 5× One Taq Standard Reaction buffer, 1.25 U One Taq Hot Start DNA polymerase (Thermo Scientific), and 100 ng template genomic DNA. PCR reactions were performed in a programmable thermal cycler (MultiGene OptiMax, Labnet International, Whitehead Scientific) with an initial denaturation step (94°C, 30 s), followed by 30 cycles of denaturation (94°C, 30 s), primer annealing, and elongation (see Table 1). Cycling was completed by a final elongation step (68°C, 10 min), followed by cooling to 4°C. The amplified fragments were purified using the DNA Clean and Concentrator™‐25 kit (Zymo Research, Inqaba Biotechnical Industries) according to the manufacturer's instructions.

**TABLE 1:** Primer sequences and PCR conditions for the amplification of the 16S rRNA gene and the housekeeping genes *rpoA, dnaA, pheS,* and *tuf*

Gene	Primer name	Primer sequence (5′ → 3′)	Annealing temp. (°C)	Elongation time (s)	Amplified fragment size (bp)	Reference
16S rRNA	27F	AGAGTTTGATCMTGGCTCAG	50	90	1,450	Lane (1991); Turner, Pryer, Miao, and Palmer (1999)
	1492R	GGTTACCTTGTTACGACTT				
*rpoA*	rpoA‐21‐F	ATGATYGARTTTGAAAAACC	46	60	800	De Bruyne et al. (2007)
	rpoA‐23‐R	ACHGTRTTRATDCCDGCRCG				
*dnaA*	dnaA445‐F	GGTGGCGTTGGTCTAGGWAAAACMCAYYTRATG	55	60	800	Chelo et al. (2007)
	dnaA1253‐R	TGCATCACAGTTGTATGATCYYKMCCRCCAAA				
	dnaA445‐Fs^a^	GGTGGCGTTGGTCTAGG				
*pheS*	pheS‐21‐F	CAYCCNGCHCGYGAYATGC	56	30	400	De Bruyne et al. (2007)
	pheS‐23‐R	GGRTGRACCATVCCNGCHCC				
*tuf*	Tuf‐for	ATGGCAGAAAAAGAACATTACG	52	90	1,200	Sarmiento‐Rubiano et al. (2010)
	Tuf‐rev	AGTAACYTGACCRGCACCAAC				

^a^ Sequencing primer only.

### 2.4 Gene sequencing and phylogenetic analyses

Partial 16S rRNA, *rpoA, dnaA*, *pheS,* and *tuf* gene sequencing was performed using BigDye Cycle Sequencing chemistry (Applied Biosystems), according to the manufacturer's instructions. Sequence similarity searches were performed using the Basic Local Alignment Search Tool (BLAST) algorithm (Altschul, Gish, Miller, Myers, & Lipman, 1990). Reference 16S rRNA, *rpoA, dnaA*, *pheS,* and *tuf* gene sequences from respective type strains with names in standing nomenclature were retrieved from the National Centre for Biotechnology Information (NCBI) (https://www.ncbi.nlm.nih.gov/) and included in the analyses. Reference strains and their species names are indicated in the respective figures. Strain numbers were omitted for reference strains in the phylogenetic trees based on concatenated gene sequences since sequences representative of the same type strains, but obtained from different culture collections, were used. GenBank accession numbers for 16S rRNA, *rpoA, dnaA*, *pheS,* and *tuf* gene sequences of representative strains for each sampling location, determined in this study, are listed in Table 2. For phylogenetic inference, seven separate alignments were created: five corresponding to the single‐locus alignment of 16S rRNA, *rpoA*, *dnaA*, *pheS,* and *tuf* genes, and two alignments corresponding to the concatenation of the housekeeping genes *rpoA‐dnaA* (for *Leuconostoc* spp.) and *pheS‐tuf* (for *Lactobacillus* spp.). Sequences were aligned with ClustalW (Thompson, Higgins, & Gibson, 1994), as implemented in the BioEdit Sequence Alignment Editor program (Hall, 1999). A data matrix for each alignment was created for the representative sequences of strains at each sampling location and sampling time. Phylogenetic analyses were conducted using the Molecular Evolutionary Genetics Analysis (MEGA) version 7.0 software (Kumar, Stecher, & Tamura, 2016). Evolutionary histories were inferred using the maximum likelihood method with the Kimura 2‐parameter model (Kimura, 1980) for 16S rRNA sequence analyses, *Lactobacillus* spp. *tuf* sequence analyses and *Lactobacillus* spp. *pheS‐tuf* concatenated sequence analyses. The Tamura 3‐parameter model (Tamura, 1992) was used for respective *Leuconostoc* spp. *rpoA, dnaA,* and *rpoA‐dnaA* concatenated sequence analyses and *Lactobacillus* spp. *pheS* sequence analyses. The strengths of the internal branches of the resultant trees were statistically evaluated by bootstrap analysis (Felsenstein, 1985) with 100 bootstrap replications.

**TABLE 2:** GenBank accession numbers of the sequences as determined in this study for representative *Leuconostoc* and *Lactobacillus* strains for each sampling location

Strain ID	16S rRNA	*rpoA*	*dnaA*	*pheS*	*tuf*
A2‐5	MK673936	MK679630	‐	‐	‐
A2‐6	MK673937	MK679631	MK679647	‐	‐
A16‐8	MK673938	MK679632	‐	‐	‐
A16‐9	MK673939	MK679633	MK679641	‐	‐
A19‐15	MK673940	MK679634	MK679642	‐	‐
A19‐37	MK673941	MK679635	‐	‐	‐
B1‐23	MK673942	MK679636	MK679643	‐	‐
B9‐3	MK673943	MK679637	MK679644	‐	‐
B9‐41	MK673944	MK679638	‐	‐	‐
B16‐2	MK673945	MK679639	MK679645	‐	‐
B19‐1	MK673946	MK679610	MK679646	‐	‐
A2‐7	MK673947	‐	‐	MK679648	MK679654
A9‐3	MK673948	‐	‐	MK679649	MK679655
A19‐103	MK673949	‐	‐	MK679650	MK679656
B2‐4	MK673950	‐	‐	MK679651	MK679657
B9‐17	MK673951	‐	‐	MK679652	MK679658
B19‐10	MK673952	‐	‐	MK679653	MK679659

## 3 RESULTS

### 3.1 16S‐rRNA gene sequence analysis of *Leuconostoc* and *Lactobacillus* spp.

**FIGURE 1:**
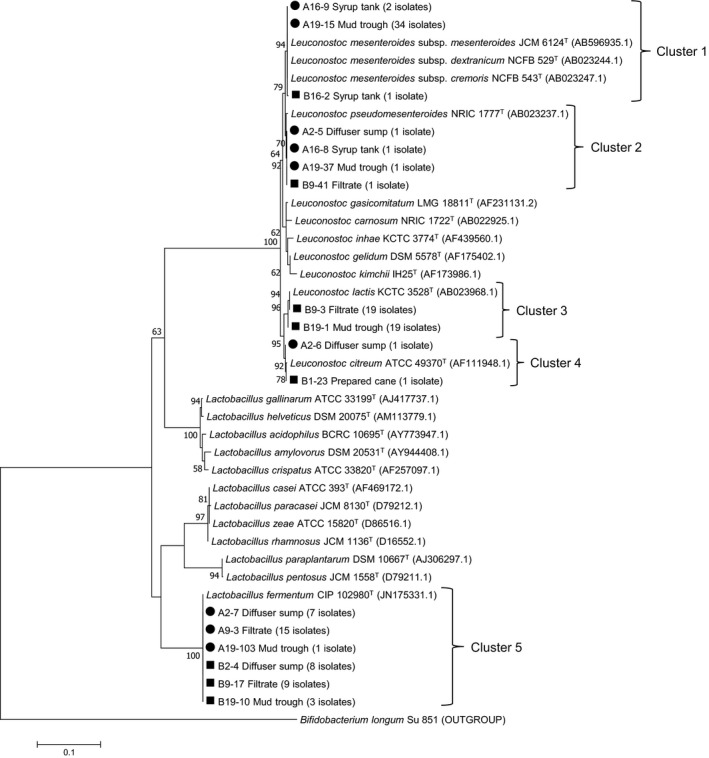
Phylogenetic tree based on partial 16S rRNA gene sequences of *Leuconostoc* and *Lactobacillus* species isolated from five sampling points in a South African sugarcane processing factory. Isolates from the sampling time when low‐dextran content was observed in raw sugar are labeled with a circle (●) and those when high dextran in raw sugar was reported with a square (■). The tree was constructed using the maximum likelihood method with MEGA 7.0 software, and representative isolates from each sampling point are shown, with the number of isolates indicated in brackets. Sequence data of reference strains were from GenBank. Genetic distances were computed by Kimura's 2‐parameter model (Kimura, 1980). The final dataset had a total of 897 positions. Bootstrap values over 50% (based on 100 replications) are shown at each node. Bar, % estimated substitution per nucleotide position. *Bifidobacterium longum* Su 851 was used as the outgroup

One‐hundred and twenty‐four isolates of *Leuconostoc* and *Lactobacillus* spp. grouped into five distinct clusters based on 16S rRNA gene sequence analyses (Figure 1). Of the 124 isolates, 81 were classified as members of the genus *Leuconostoc*. Thirty‐seven isolates formed a tight group with the type strains of *Leuc. mesenteroides* subsp. *mesenteroides* (JCM 6124^T^), *Leuc. mesenteroides* subsp. *dextranicum* (NCFB 529^T^), and *Leuc. mesenteroides* subsp. *cremoris* (NCFB 543^T^) in Cluster 1. Four isolates grouped with the type strain of *Leuc. pseudomesenteroides* (NRIC 1777^T^) in Cluster 2. Cluster 3 was the largest, with 38 isolates phylogenetically closely related (similarity values ranging 99.8%–99.9%) to the type strain of *Leuconostoc lactis* (KCTC 3528^T^). Two isolates grouped with the type strain of *Leuconostoc citreum* (ATCC 49370^T^) in Cluster 4. All 43 isolates preliminary identified as members of the genus *Lactobacillus* grouped with the type strain of *Lactobacillus fermentum* (CIP 102980^T^) in Cluster 5.

### 3.2 Phylogenetic analyses of amplified *rpoA*, *dnaA*, and *rpoA*‐*dnaA* concatenated gene sequences of *Leuconostoc* spp.

**FIGURE 2:**
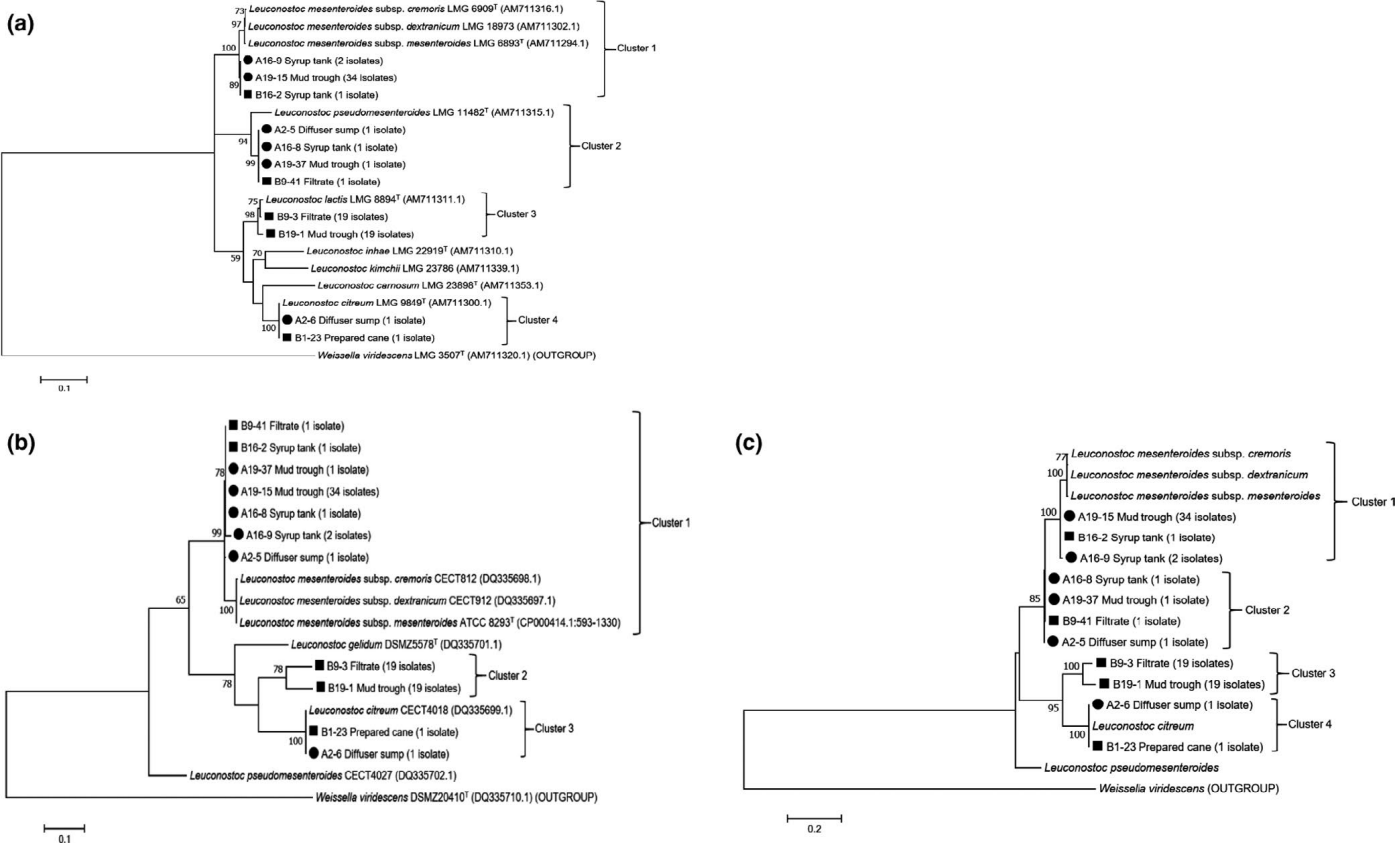
Phylogenetic trees based on partial *rpoA* (a), *dnaA* (b), and *rpoA‐dnaA* concatenated (c) gene sequences of *Leuconostoc* species isolated from five sampling points in a South African sugarcane processing factory. Isolates from the sampling time when low‐dextran content was observed in raw sugar are labeled with a circle (●) and those when high dextran in raw sugar was reported with a square (■). The tree was constructed using the maximum likelihood method with MEGA 7.0 software, and representative isolates from each sampling point are shown, with the number of isolates indicated in brackets. Sequence data of reference strains were from GenBank. Bootstrap values over 50% (based on 100 replications) are shown at each node. Bar, % estimated substitution per nucleotide position

The *Leuconostoc* isolates (81 in total) grouped into four clusters based on partial *rpoA* gene sequence analysis (Figure 2a). In accordance with 16S rRNA gene sequence analyses, 37 isolates were phylogenetically related to *Leuc. mesenteroides* subspp*. mesenteroides, dextranicum,* and *cremoris*, with similarity values ranging from 98.3% to 98.5% (Figure 2a). Four isolates were phylogenetically related to *Leuc. pseudomesenteroides* (similarity value of 95.0%), 38 to *Leuc. lactis* (similarity values ranging 98.3%–99.3%), and two to *Leuc. citreum* (100% similar) (Figure 2a).

The phylogenetic tree inferred from partial *dnaA* sequence analyses of *Leuconostoc* isolates is shown in Figure 2b. Eighty‐one isolates grouped into three clusters. Cluster 1 contained 41 isolates related to *Leuc. mesenteroides* subspp. *mesenteroides, dextranicum,* and *cremoris*; Cluster 2 hosted 38 isolates which previously clustered with *Leuc. lactis* 16S rRNA and *rpoA* gene sequences (Figures 1 and 2a), and Cluster 3 contained two isolates that grouped with *Leuc. citreum*. A reference sequence for the *dnaA* gene from *Leuc. lactis* was not available from GenBank. However, based on the 16S rRNA and *rpoA* gene sequence results, it is suggested that Cluster 2 (Figure 2b) represents *dnaA* gene sequences of bacteria phylogenetically related to *Leuc. lactis*. The phylogeny obtained for *dnaA* sequence analysis (Figure 2b) is in disagreement with the phylogeny of the trees inferred from 16S rRNA and *rpoA* sequence analyses (Figures 1 and 2a) for isolates A2‐5, A16‐8, A19‐37, and B9‐41, which previously clustered with *Leuc. pseudomesenteroides*. Based on *dnaA* sequences, these four isolates are related to *Leuc. mesenteroides* subspp. *mesenteroides, dextranicum,* or *cremoris* (Figure 2b). A higher phylogenetic resolution may be obtained for these isolates by the analyses of additional housekeeping genes such as *atpA* (encoding alpha subunit of ATP synthase) or *pheS* (encoding phenylalanyl‐tRNA synthase) (De Bruyne et al., 2007).

None of the loci examined (Figures 1 and 2a,b), nor the concatenated *rpoA‐dnaA* sequences (Figure 2c), allowed discrimination between subspecies within *Leuc. mesenteroides*.

### 3.3 Phylogenetic analyses of amplified *pheS*, *tuf*, and *pheS*‐*tuf* concatenated gene sequences of *Lactobacillus* spp.

**FIGURE 3:**
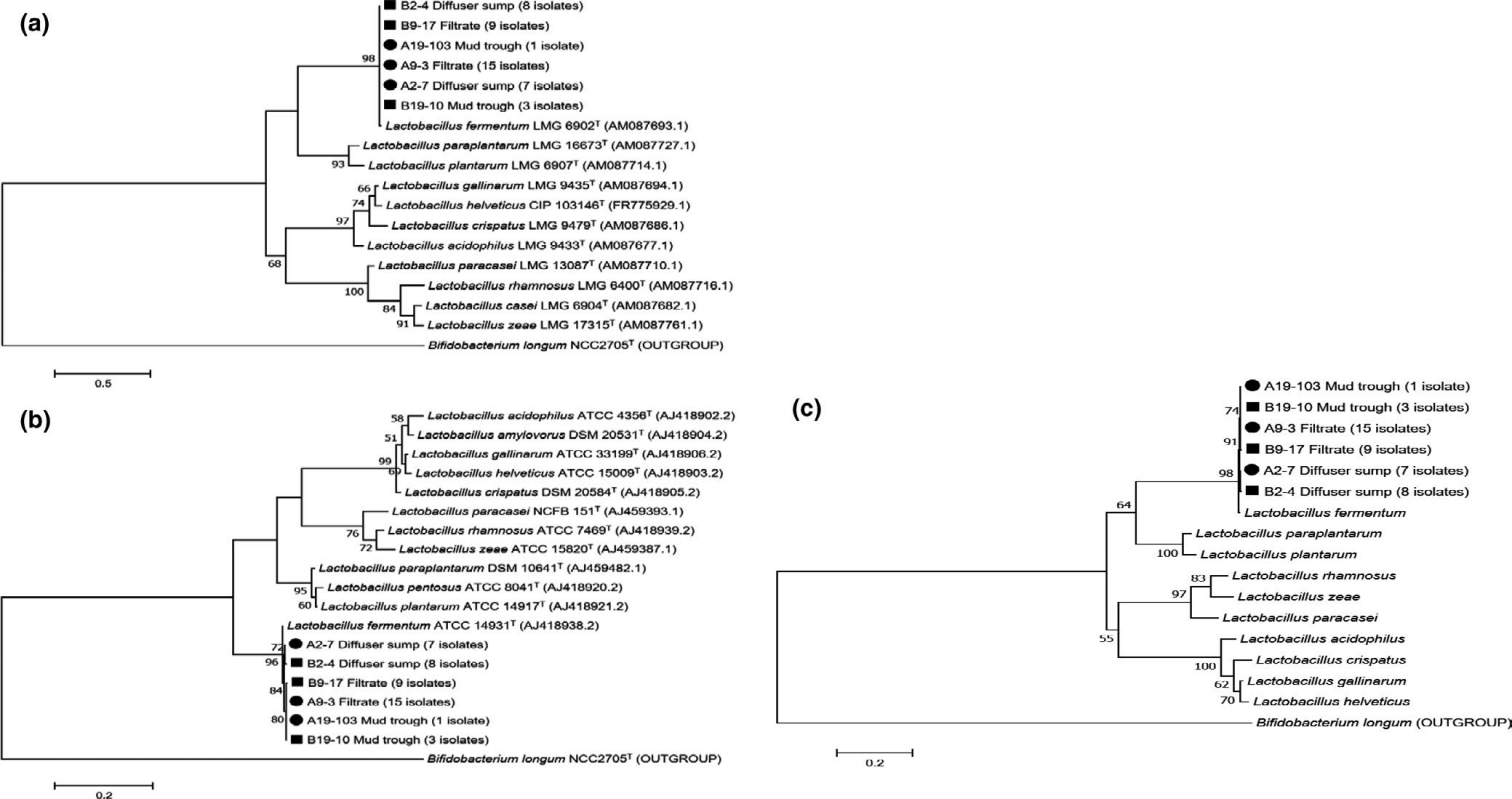
Phylogenetic trees based on partial *pheS* (a), *tuf* (b), and *pheS‐tuf* concatenated (c) gene sequences of *Lactobacillus* species isolated from three sampling points in a South African sugarcane processing factory. Isolates from the sampling time when low‐dextran content was observed in raw sugar are labeled with a circle (●) and those when high dextran in raw sugar was reported with a square (■). The tree was constructed using the maximum likelihood method with MEGA 7.0 software, and representative isolates from each sampling point are shown, with the number of isolates indicated in brackets. Bootstrap values over 50% (based on 100 replications) are shown at each node. Bar, % estimated substitution per nucleotide position

The 43 lactobacilli in this study clustered with *L. fermentum* in the phylogenetic trees inferred from partial *pheS* (Figure 3a), *tuf* (Figure 3b), and *pheS‐tuf* concatenated (Figure 3c) gene sequences, with high bootstrap support in all three trees. This is in agreement with the clustering obtained from partial 16S rRNA gene sequence analyses (Figure 1).

## 4 DISCUSSION

The *rpoA* housekeeping gene has previously been successfully applied for the phylogenetic resolution of *Leuconostoc* spp. (De Bruyne et al., 2007; Rahkila, De Bruyne, Johansson, Vandamme, & Björkroth, 2014). Although strains of *Leuconostoc* spp. could be differentiated to species level by phylogenetic analyses of their *rpoA* housekeeping genes, the phylogenetic resolution was not higher compared to 16S rRNA gene analyses. Comparison of *dnaA* gene sequences differentiated isolates that clustered with the type strains of *Leuc. mesenteroides* and *Leuc. citreum*. None of the genes analyzed differentiated between subspecies of *Leuc. mesenteroides*. Reasons for this have been ascribed to an inadequate number of available genomes and genomic diversity studies and fewer strains of *Leuc. mesenteroides* subspp. *cremoris*, *dextranicum,* and *jonggajibkimchii* studied compared *Leuc. mesenteroides* subsp. *mesenteroides* (Ricciardi et al., 2020). Only a few strains of *Leuc. mesenteroides* subspp. *cremoris* and *dextranicum* are listed in microbial culture collections (*e.g.,* ATCC, DSMZ, LMG), and for some of them, the isolation source is unknown, which limits information on strain diversity. The description of *Leuc. mesenteroides* subsp. *jonggajibkimchii* was based on phenotypic and genomic features of a single isolate (DRC1506^T^, Jeon et al., 2017). Ricciardi et al. (2020) suggested that more strains need to be studied to reach a confident separation at the subspecies level (Ricciardi et al., 2020). For this reason, *Leuc. mesenteroides* subsp. *jonggajibkimchii* was not included in any of the phylogenetic analyses presented in this work. Ricciardi et al. (2020) used multiplex PCR to differentiate dextran‐producing strains of *Leuc. mesenteroides* subsp. *mesenteroides* from nondextran producing strains of *Leuc. mesenteroides* subsp. *cremoris*. The method was based on the detection of L‐arabinose isomerase, dextransucrase, and PTS‐sorbose‐transporter subunit IIC genes. Although multiplex PCR could not resolve ambiguous identification of *Leuc. mesenteroides* subspp. *dextranicum* and *jonggajibkimchii* strains, the method may be of value to identify gum‐producing *Leuconostoc* spp. isolated from sugarcane processing factories.

The phylogenetic analyses of *pheS* (encoding phenylalanyl‐tRNA synthase) and *tuf* (encoding elongation factor Tu) genes have proven to be a valuable tool for the taxonomic resolution of *Lactobacillus* spp. (Chavagnat, Haueter, Jimeno, & Casey, 2002; Naser et al., 2007; Sarmiento‐Rubiano et al., 2010; Ventura, Canchaya, Meylan, Klaenhammer, & Zink, 2003; Yu et al., 2012). In this study, single‐locus analysis, as well as a concatenation of the *pheS* and *tuf* housekeeping gene sequences, yielded identical phylogenies for the *Lactobacillus* isolates corresponding to *L. fermentum*. This was in agreement with the 16S rRNA gene sequence analysis.

The quality of sugarcane reaching the factory has to be tested regularly. This is a major challenge since there is no rapid, reliable, and inexpensive method available to detect the level of cane deterioration (Eggleston & Harper, 2006). The modified haze method (Anon, 2015), used by the sugarcane processing factory samples were taken from, determines the overall gum content in raw sugar and not dextran. In this study, we have analyzed the sequences of housekeeping genes to identify dextran‐producing strains of *Leuconostoc* and *Lactobacillus* spp. from various locations in the sugarcane processing factory. The presence of dextran‐producing strains served as an indicator of dextran production and cane deterioration. *Leuconostoc* spp. and *L. fermentum* produce dextran and other metabolic products, including mannitol, lactic and acetic acids, and ethanol (Daeschel, Andersson, & Fleming, 1987; Eggleston, Legendre, & Tew, 2004), and are thus considered spoilage organisms. Although dextran is considered to be the most detrimental product to the factory because it is a high‐viscosity polymer, *Leuconostoc* and *Lactobacillus* spp. are also capable of producing other polymers such as levan and alternan (Dutta, Das, & Goyal, 2012; Kralj et al., 2004; Naessens, Cerdobbel, Soetaert, & Vandamme, 2005). The formation of these polymers may be underestimated as contributors to impeding high‐viscosity problems in sugarcane processing, mainly because of the nonspecific nature of the dextran quantification method (Anon, 2015) used in the sugar industry. These bacterial metabolites may have a severe impact on the quality and quantity of produced sugar.

The number of *Leuconostoc* and *Lactobacillus* bacteria which were isolated when low‐dextran raw sugar was produced (40 and 23 isolates, respectively) was similar to the numbers of these bacteria when high‐dextran raw sugar was produced (41 and 20 isolates, respectively). Contrary to previous reports (Egan, 1966; Solomon, 2000), *Leuconostoc* spp. were not the major gum‐producing bacteria isolated from sugarcane (Nel et al., 2019a). This study showed that the isolate closely related to *Leuc. citreum* was the only *Leuconostoc* bacterium isolated from shredded (prepared) sugarcane, and it was shown previously (Nel et al., 2019b) that *W. confusa* and *W. cibaria* were the most prevalent gum‐producing bacteria on the prepared cane. Similar numbers of isolates clustering closely with *L. fermentum* were isolated from the diffuser sump, filtrate, and mud trough at both sampling times, respectively. Isolates that were phylogenetically related *Leuc. lactis* were the most prevalent in the filtrate at a time when high‐dextran raw sugar was produced. The majority of bacteria isolated from the mud belonged to species clustering with *Leuc. mesenteroides* (low‐dextran raw sugar) and *Leuc. lactis* (high‐dextran raw sugar), and isolates related to *Leuc. mesenteroides* were the dominant bacteria isolated from the syrup tank at both sampling times.

Correct process control, especially of high‐temperature streams, is critical to prevent microbial growth in a sugarcane processing factory. In this study, filtrate temperatures of 58°C and 29°C were recorded when sampled at times of low and high‐dextran concentrations in raw sugar, respectively. Filtrate temperatures are usually around 60°C. Factory staff acknowledged that the low filtrate temperature recorded during the second sampling was due to a processing error. At this time, strains of *Leuc. lactis* and *L. fermentum* were isolated. *Lactobacillus fermentum* dominated the filtrate sample taken at the first sampling. The temperature of the mud in the mud trough at this time (35°C) was much lower compared to the second sampling (64°C), possibly due to stoppages and longer retention times of the mud in the trough, resulting in cooling of the mud. A considerable number of *Leuc. mesenteroides* strains (31% of the total number of strains isolated during the first sampling) were from mud at 35°C. On the contrary, *Leuc. lactis* was the major gum‐producer in the mud during the second sampling when the temperature was higher (64°C). *Leuc. lactis* has a higher heat resistance than *Leuc. mesenteroides* (Logan & De Vos, 2009). Although the filtrate is recirculated to the mixed juice tank, none of the gum‐producing bacteria isolated in the filtrate were detected in the juice sampled from the mixed juice tank. This is presumably due to the high temperatures (67°C and 73°C, respectively) recorded for juice samples, which allowed the growth of endospore‐forming *Bacillus* species, but not *Leuconostoc* and *Lactobacillus* spp. (Berendsen et al., 2016; Logan & De Vos, 2009; Warth, 1978).

## 5 CONCLUSIONS

Dextran is an unwanted bacterial metabolite in sugarcane processing, leading to reduced factory throughput, quality, and quantity of the produced sugar. This study showed that the number of lactobacilli at the various locations at both sampling times was similar and all species were found to be related to *L. fermentum*. However, the diversity of the leuconostocs was found to vary depending on the temperature of the location from which they were isolated. Correct process control of high‐temperature factory streams is therefore critical to limit microbial growth and gum‐formation in a sugarcane processing factory.

The phylogenetic relationships, based on housekeeping gene sequence analyses, of *Leuconostoc* and *Lactobacillus* species isolated from various unit operations of a sugarcane processing factory were established at a time when low‐ and high‐dextran raw sugar, respectively, were produced. Analyses of *rpoA* sequences proved as effective as 16S rRNA gene sequence analyses to determine the phylogenetic relationships between *Leuconostoc* spp. isolated from sugarcane. Comparison of *dnaA* sequences differentiated isolates that clustered with the type strains of *Leuc. mesenteroides* and *Leuc. citreum*. Clear differences were recorded between *Lactobacillus* spp. and the type strains of *Lactobacillus* spp. when sequences of *pheS* and *tuf* were compared. Although the housekeeping genes did not prove more discriminating compared to 16S rRNA gene sequence analyses, this study illustrated the potential of gene‐based methods as an alternative to phenotypic methods to differentiate lactic acid bacteria in the sugarcane industry.

## CONFLICT OF INTEREST

None declared.

## AUTHOR CONTRIBUTIONS

**Sanet Nel:** Conceptualization (equal); Formal analysis (equal); Investigation (lead); Methodology (equal); Writing‐original draft (equal). **Stephen B. Davis:** Conceptualization (equal); Supervision (equal); Writing‐review & editing (equal). **Akihito Endo:** Supervision (equal); Writing‐review & editing (equal). **Leon M. T. Dicks:** Supervision (equal); Writing‐review & editing (equal).

## ETHICS STATEMENT

None required.

## Data Availability

All data are provided in full in the results section of this paper. The relevant DNA sequences were deposited in GenBank, and accession numbers are listed in Table 2.
